# Strangulated Hernia in Early Infancy

**Published:** 1901-06-08

**Authors:** 


### STRANGULATED HERNIA IN EARLY INFANCY.

A case of successful operation for strangulated hernia in a premature child only five weeks old is recorded by Mr. D'Arcy Power.[1] The patient, a very wasted and ill-nourished male child, aged five weeks, and said to have been prematurely born at seven months, was admitted into St. Bartholomew's Hospital under the care of Mr. D'Arcy Power on April 15th. A swelling was first noticed in the left groin on the 12th. On the 13th the child had vomited every time he was put to the breast, and had cried all day. The bowels had not been open since the morning of the 12th. He was blue and collapsed, and in the groin there was a hernial swelling as large as a walnut. As this could not be reduced it was operated on. At the operation the hernia was found to be of the congenital variety, there was practically no fluid in the sac, and this contained a knuckle of intestine, rather deeply congested and a very little piece of omentum. The stricture, very tight and situated at the internal ring, was divided, the bowel returned, and the sac isolated, ligatured in two places, and divided, the lower part being left to form a tunica vaginalis, while the upper portion was stitched to the peritoneal surface of the abdominal wall. The external abdominal ring was closed with a single suture. All went on well, and on the sutures being removed on the fourth day the wound was found to be perfectly healed. The child weighed 4 pounds 12 ounces on admission, and 5 pounds 1 ounce when he left the hospital on April 30th. Curiously enough, the same child was taken to Mr. Power as a new patient at the Metropolitan Dispensary on May 21st for the relief of a hernia, this time on the right side, and as this appeared to be due to straining caused by a very tight phimosis, a circumcision was performed, from which the child recovered with a single dressing; so that, as an example in clinical surgery, this puny infant commenced life with considerable *éclat*. Strangulated hernia in such young children is very rare in comparison with the very large number of cases in which hernia exists. About a hundred cases of herniotomy for strangulation in children under one year old have been recorded, about sixteen of which occurred during the first month and the same number during the second.

[1] Lancet, June

